# Plasma 5-miRNA as Biomarkers for Identifying Prostate Cancer Patients

**Published:** 2019-09

**Authors:** Mojtaba SAFFARI, Mohammad Hossein MODARRESSI, Reza SHIRKOOHI, Bita HASSANI, Mandana AFSHARPAD, Gholamreza RAFIEI, Gholamreza ABROODI, Mir Saeed YEKANINEJAD, Jamal ARFAEE, Seyed Mohammad AKRAMI

**Affiliations:** 1. Department of Medical Genetics, School of Medicine, Tehran University of Medical Sciences, Tehran, Iran; 2. Cancer Biology Research Center, Cancer Institute of Iran, Tehran, Iran; 3. Cancer Research Center, Cancer Institute of Iran, Tehran University of Medical Sciences, Tehran, Iran; 4. Department of Basic Sciences, Maybod Branch, Islamic Azad University, Yazd, Iran; 5. Department of Epidemiology and Biostatistics, School of Public Health, Tehran University of Medical Sciences, Tehran, Iran; 6. Department of Basic Sciences and Medicine, Zanjan Branch, Islamic Azad University, Zanjan, Iran

## Dear Editor-in-Chief

To find a non-invasive tumor biomarker for diagnosis and/or prognosis is currently one of the most rapidly growing areas in cancer research. The stability of miRNAs in body fluids has been caused that they are considered as a novel class of biomarkers (1). In this study, we investigated the potential of circulating miRNAs as novel biomarkers in serum patients with prostate cancer. Although there are proteins as biomarkers their measurement is not easy performed in the clinical diagnosis and also sensitivity or specificity of them is not very high. Serum miRNAs are stable and can be indicated in serum; therefore it is very much facilitating the clinical use of such tests (2). It has been previously identified as a subset of microRNAs expressed in tumor prostate tissues versus normal tissues. Interestingly, our observation suggests that deregulation of all of the indicator miRNA (miR-let7a & b, miR-124, miR-146a, and miR-185) in cancerous samples versus BPH and non-cancerous samples. These miRNAs may serve as repression of progression of tumors and or function as tumor suppressor or reflect tissue differentiation. Now, usually, only individuals with PSA increased values are referred to invasive diagnostic tests. Therefore, we suggested that the observed differential miRNA expression pattern differentiated Gleason scores >7 & ≤7 prostate cancer groups from BPH and non-cancerous groups. The miR-124a, miR-146a & b, miR-185, miR-16 and let-7a & b have significant differential expression in normal prostate tissue compared cancer tissues and the expression levels of these microRNAs, independently of the PSA level, were highly suggestive of prostate cancer (3).

There is contradictoriness in the reported results of miR-let7a expression level in prostate cancer versus normal samples. For example, miR-let7a upregulated 1.53-fold in tumor tissues, however; others demonstrated family miR-let7 expression is decreased in prostate cancer. Cell growth was blocked at the G0-G1 phase by downregulating of mRNA expression of *E2F2* and *CCND2* in prostate cancer. Down-regulation of let-7b may be correlated with aggressive cancer characteristics. In prostate cancer, miR-146a has shown to suppress *ROCK1* oncogene and inhibit cell growth and induce apoptosis in miR-146a-ROCK1-caspase3 pathway and suppressed EMT phenomena (4). Therefore, miR-let7a may be as oncogene suppressor and the recent findings suggest that let-7a and miR-146a may be a potential biomarker and novel therapeutic candidate in prostate cancer. MiR-124 is a tumor suppressor microRNA that caused to inhibit growth of cancer prostate and induce apoptosis by targeting androgen receptor gene. Our study has shown the relative expression level of miR-124 in the cancerous sample (Gleason scores >7≤) was reduced. The miR-124 as a putative tumor suppressor was down-regulated in prostate cancer and caused overexpression of talin-1 in prostate cancer. miR-124 revealed anti-attachment and anti-migration (5). MiR-185 operates as a tumor suppressor in various cancers. miR-185 is decreased and inhibits progression of tumor through direct down-regulation of AR expression (6).

**Fig. 1:**
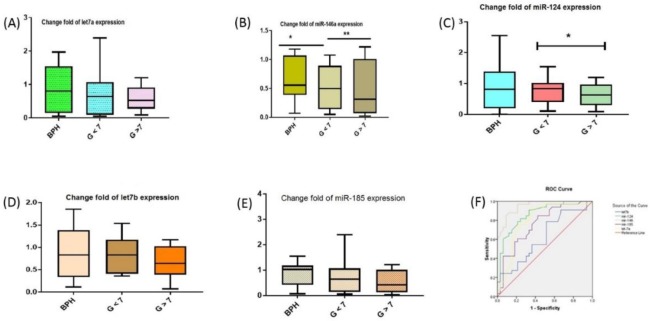
(A–E) MiRNAs expression in four groups of our study was measured by RT-qPCR. Values represent means and error bars represent the SEM. (*P<0.05, **P<0.01). (F) The ROC curves of the 5 miRNAs

Our data has shown that the expression level of miR-185 in prostate cancer samples lower than in control samples (Fig. 1). ROC curve analysis was done to evaluate the accuracy of the 6 miRNAs in the plasma between two groups case (cancerous sample with Gleason scores >7≤) and control (BPH and non-cancerous). The AUCs and 95% CI for miR-let7a /b, miR-124, miR-146a and miR-185 is shown in Table 1.

**Table 1:** The data ROC area of miRNA

***Name of miRNA***	***ROC area***	***95% CI***	***Sensitivity (%)***	***Specificity (%)***
Let-7a	0.799	0.686–0.913	87	67
Let-7b	0.622	0.489–0.758	80	32
miR-124	0.764	0.637–0.890	85	70
miR-146	0.952	0.907–0.996	87	88
miR-185	0.760	0.644–0.876	88	55

Different significant expression levels of miRNA were shown between the compared groups cause to further test our miRNA pattern on larger and independent groups for their use as diagnostic or prognostic substitute biomarkers in patients with high PSA serum levels. Nonetheless, many issues have to be considered before these data can be translated into a clinically useful as non-invasive screening strategy for prostate cancer patients.
